# Are Scientists Sufficiently Ambitious? Season 2

**DOI:** 10.1093/function/zqad032

**Published:** 2023-06-16

**Authors:** Ole H Petersen

**Affiliations:** School of Biosciences, Sir Martin Evans Building, Cardiff University, Wales CF10 3AX, UK

**Keywords:** research grants, research assessment, research funding models, impact factors, useful knowledge, mental health crisis

The article “Are scientists sufficiently ambitious?”^1^ is by far the most downloaded editorial so far published in *Function*, and I have had more personal reactions to this piece than to any other item presented in the journal. The reactions have overwhelmingly been positive, since most colleagues agree that the problems highlighted in the editorial are critically important. However, some correspondents have also made the point that it is not sufficient to identify the problems. Solutions are needed. Hence, I am now revisiting the theme, to propose ways forward that could rectify an increasingly difficult situation.

One of the main problems identified in the original editorial was the current funding system that encourages generation of more and more data, resulting in biologists “drowning in a sea of data and starving for knowledge.”^2^ Because it is more expensive to generate new data than to provide context, models, and theories and because Universities increasingly evaluate staff on the basis of how much money they bring into the institution (overheads are essential for the sustainability of Universities), scientists are chasing research grants at the expense of most other activities. Unfortunately, this activity has become increasingly time consuming, not least because the probability of rejection is high. Many applications are written to ensure that at least a few are successful. Even for those who manage to secure funding, grant writing is taking up far too much time, and for the many who fail, it is of course a waste of time. Furthermore, it is not only the applicants who lose valuable time. The elaborate evaluation system, which is deemed necessary to select the “best” proposals, takes away time from many experienced scientists, who serve as reviewers and grant panel members, time that could have been used for primary research activities.

One of the reasons for the declining success rates for grant applications is the substantial rise in the number of applicants. This is ultimately due to increasing numbers of PhD students, postdoctoral research fellows, and Principal Investigators (PIs). Perhaps PIs should take fewer but more able and committed graduate students, reducing the unnecessary amount of “useless” data and uncited papers. Unfortunately, this would be opposed by Universities, since they benefit significantly from fees and tuition costs.

Many would argue that the peer review of project or program grant applications and the associated competitive ranking are well-established procedures without which an effective research funding system cannot work. However, the German Max Planck Society, for example, operates in a different way. Directors at Max Planck Institutes are assured of funding throughout their tenure and can pursue their interests without having to make specific project/program applications. Each institute has an external advisory board but, based on my own experience of having served on such boards for many years, these bodies are not very intrusive and basically, in their reports to the President of the Society, highlight areas in which there has been spectacular progress, and where additional support may be desirable. Importantly, the advisory boards cannot prevent a Scientific Director from pursuing his/her specific interests even if they don’t agree with the “direction of travel.” The important decisions are made when Directors are appointed. Thereafter, the long-term support frees Directors from short-term tactical considerations and encourages the pursuit of ambitious goals that may require investment over many years before success is achieved. In contrast, the project/program grant model inevitably encourages the pursuit of “low-hanging fruit,” because of the need to publish quickly to secure the next grant award.

Because most research grants are short term, evaluation of merit is also short term. There is a premium for publishing quickly in the so-called high-impact journals, but often no reward for long-term utility. Thus, we have masses of papers with enormous amounts of new data that do not form the basis for useful exploitation, are hardly cited by anybody and therefore not used.

Nevertheless, our research system is not broken. Enormous progress in the form of new insights that have had real impact on understanding, prevention, and therapy of diseases have been made, but that does not mean that our system functions optimally. We do not know, for example, whether the current grant evaluation system is better than random allocation! It is generally assumed that it must be, but do we have evidence for this? A couple of years ago, when seated next to the Chair of one of the world’s largest research funding organizations at a dinner, I asked this question. The answer was, as I had expected, that we don’t have such evidence. It may be impossible to obtain it. Inevitably, those who succeed in the competition for grants will be more successful than those who fail to obtain grants. We’ll never know whether those who failed would have done as well as the successful ones if they had been lucky enough to receive funding.

Perhaps it is time to conduct experiments to test the validity of the current very expensive grant evaluation system. Research funding bodies could set aside a part of their funds for random allocation. Certain entry levels and checks could be required, ensuring that nonsense applications were excluded. Such an experiment would have to be conducted on a large scale and over many years, as only time would tell whether the outcomes of the two very different funding allocation models were different and, if so, which one was superior. In any case, this approach would highlight that receiving a research grant is not in itself an achievement. It merely enables work to be done. If useful knowledge emerges from the work, then we can talk about an achievement. If, on the other hand, work based on a research grant does not lead to useful knowledge, then resources have been wasted and this should not be rewarded.

It takes time before it is possible to evaluate research achievements and we should therefore be more patient and reduce the frequency of such assessments. This would free up valuable time for everyone and therefore increase the overall efficiency of the system. The current research assessment system is not sustainable and only appears to be working because many evaluators “cut corners.” Although DORA (Declaration of Research Assessment) dictates that evaluation of research outputs must not be based on the IF (Impact Factor) of the journal in which they are published, the reality is unfortunately quite different, although this will—naturally—always be denied. The reason is simply that assessment by journal IF is by far the quickest way to rank a research article in the first years after publication. It is telling that, as Editor of *Function*, the one question I am constantly asked is: “What will be *Function*’s first IF?”

The enormous pressure on individual scientists to publish in high-impact journals and thereby secure large research grants is undoubtedly one of several drivers behind the current mental health crisis in science.^3^ “The advent of “metrics” has, in many ways, distorted the whole publication process as well as the assessment of individual academic merit.”^4^ “The whole system needs a massive overhaul.”^3^

## Data Availability

There are no data in this editorial.
